# Perception and Impact of White Spot Lesions in Young People Undergoing Orthodontic Treatment and Their Guardians: Protocol for a Mixed Methods Study

**DOI:** 10.2196/60213

**Published:** 2025-09-12

**Authors:** Amaar Obaid Hassan, Janine Doughty, Jayne Harrison

**Affiliations:** 1 Department of Orthodontics, School of Dentistry, Liverpool University, Pembroke Place, Liverpool, L3 5PS, United Kingdom; 2 School of Dentistry, University of Liverpool, Liverpool, United Kingdom; 3 Orthodontic Department, Liverpool University Dental Hospital, Liverpool, United Kingdom

**Keywords:** orthodontics, white spot lesions, fixed appliances, dentistry

## Abstract

**Background:**

White spot lesions (WSLs) are white marks that can form on teeth during orthodontic treatment with fixed appliances and become apparent once they are removed. About half of people who have fixed appliance treatment get WSLs. They are usually caused by poor toothbrushing around the brace. Although there have been studies that have investigated the prevention and treatment of WSL, there remain uncertainties about what young people and their parents or guardians know or feel about them. A Cochrane review concluded that patient-reported outcomes have been overlooked in WSL prevention studies.

**Objective:**

The aim of this study is to explore young people’s and their parents’/guardians’ perceptions, attitudes, and feelings toward WSLs using a mixed methods study.

**Methods:**

This is a mixed methods study. Part 1 is a cross-sectional survey using a web-based survey questionnaire and images of pretreatment malocclusions and postorthodontic WSLs of varying severity (mild, moderate, severe). Part 2 will involve one-to-one, semistructured interviews, using open-ended questions with young people and their parents/guardians. Participants will be recruited from patients aged 11‐15 years before, during, or after undergoing orthodontic treatment at Liverpool University Dental Hospital and their parents/guardians. Part 1 (quantitative) will use a Likert scale with the option of free text comments. Data will be analyzed using descriptive statistics. Agreement between participants will be analyzed using the κ statistic. Part 2 (qualitative) will be analyzed using a modified framework analysis approach; the outcomes will be presented as themes. Transcripts from the qualitative interview will be analyzed using inductive thematic analysis. Once the qualitative and quantitative data have been analyzed, we will combine the two datasets and compare them for convergence or divergence. We will aim for a sample size of at least 100 participant and parent/guardian pairs for Part 1 and 30 interviewees for Part 2. Ethical approval was granted in November 2024. The Sponsor Permission to Proceed notification was received in January 2025.

**Results:**

Funding for the study was secured in May 2024. Recruitment started on February 2, 2025. As of August 31st, 2025, seventy five participant pairs have been recruited.

**Conclusions:**

The study will increase understanding of the impact WSLs have on oral health–related quality of life and the decision-making of young people and their parents/guardians.

## Introduction

### Background

White spot lesions (WSLs) are enamel defects that commonly appear as opaque, white, matte, chalky, or brown spots on teeth and can form around fixed orthodontic appliances. Approximately half of patients undergoing orthodontic treatment with fixed appliances will experience WSLs [1]. WSLs are caused by the combination of poor toothbrushing around the brackets of fixed appliances and frequent sugar/acidic attacks. A susceptible tooth surface exposed to bacterial plaque accumulation and fermentable carbohydrates over a sufficient period of time will undergo demineralization and potentially develop WSLs [2].

WSLs can reduce the quality and amount of enamel, leaving teeth vulnerable to damage by dental caries, thereby diminishing their lifetime prognosis [3]. Anterior surfaces on maxillary teeth are frequently affected (36%), thus WSLs may be highly visible when smiling or speaking [4].

Esthetic defects caused by WSLs may expose young people to oral health–related stigma and discrimination (eg, bullying or teasing) and impact self-esteem [5]. Low self-esteem is associated with lower grades at school, depression, and impaired social interaction with others [5]. Visible differences in dentofacial features such as tooth shape or color have been implicated as a driver for self-harm in teenagers [6]. Therefore, this study is not only important for preventing WSLs, but also for understanding the impact of WSLs on the oral health–related quality of life of young people.

There is evidence to suggest that fluoride can prevent WSLs by enhancing remineralization. Fluoride can be applied using various vehicles (eg, toothpaste, mouthwash, fluoride varnish, and casein phosphopeptides-amorphous calcium phosphate). High-strength fluoride toothpaste (5000 ppm) may be advantageous for preventing WSLs when compared with standard concentrations of fluoride toothpaste; however, these prescription-only toothpastes are only available from the age of 16 and many patients who have orthodontic treatment with fixed appliances are often below this age [7]. Once an orthodontic WSL is confirmed, then it may not be advantageous to expose it to high-strength fluoride as it can create a barrier to calcium and phosphate ions, meaning the lesion stains or persists [8].

Nonetheless, the most crucial aspect of WSL management is motivating patients to adhere to effective oral hygiene measures and noncariogenic dietary advice [9]. Clinicians believe the responsibility for preventing WSLs lies with the patient and that their postorthodontic outcomes are determined by their willingness to engage with the oral hygiene advice discussed at the commencement of treatment [10]. Visual aids have been found to be helpful to demonstrate the risks of WSLs and to motivate young people to maintain their oral hygiene [11]. A further challenge is presented by parents who are reluctant to assume responsibility for their child’s oral hygiene practices [12].

Patient-reported outcomes have been overlooked in all studies exploring the efficacy of different interventions to prevent WSLs in patients undergoing fixed orthodontic treatment [7]. Patient perceptions of WSLs can provide insights into motivators and barriers to maintaining good oral health during orthodontic treatment. Additionally, WSLs may also have cost consequences for patient and National Health Service dental services, for example, the costs of professionally applied fluoride and cosmetic restorations and their long-term maintenance.

WSLs pose an important risk for patients when considering whether to opt for orthodontic treatment [13]. At present, clinicians may be negotiating these conversations without a full evidence-based understanding of patient perceptions toward WSLs.

What remains unknown, and is the focus of our proposed study, are patients’ and parents’ perceptions of WSLs including the impact of WSLs on the acceptability of orthodontic outcomes [7]. Therefore, this study offers the opportunity for researchers to identify ways to communicate WSL risk to patients and their parents and to understand how best to motivate good oral hygiene and dietary practices during orthodontic treatment with fixed appliances.

### Reporting

Due to the absence of a well-recognized mixed methods reporting tool, we have chosen to use the Standards for Reporting Qualitative Research [14] Checklist for Reporting of Survey Studies guidelines [15] to assist with transparent and consistent reporting of each aspect of the study [16].

### Aim

The aim of this study is to explore young people’s and their parents/guardians’ perceptions of and attitudes toward WSLs.

### Objectives

The objectives of this study are to (1) create and use a questionnaire that assesses the perceptions and impact of WSLs on young people undergoing treatment with a fixed orthodontic appliance and their parents/guardians (see Multimedia Appendix 1) and (2) explore the impact and perceptions of WSL formation on young people undergoing fixed brace treatment and their parents/guardians using one-to-one interviews and visual images of malocclusions and WSLs of varying severity (see Multimedia Appendix 2).

## Methods

### Summary

**Figure 1.**
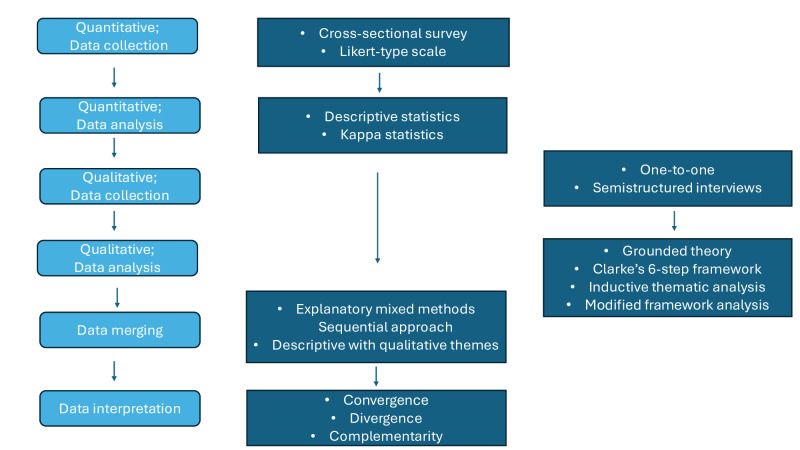
Flowchart of the mixed methods design used in this study.

A mixed methods study design was chosen for this study to provide enriched data by augmenting the quantitative findings from a questionnaire (Part 1) with data from qualitative interviews (Part 2). The questionnaire was developed in collaboration with a patient and public involvement (PPI) group. Following completion of the quantitative research (Part 1), meetings of the research team and the PPI group will be organized to review the data and develop the interview schedule for the qualitative phase (Part 2). The interview schedule will be based on the findings of Part 1 of the research. Thus, an explanatory sequential mixed methods approach will be used, whereby the qualitative data will expand on the understanding gained from the questionnaire [17]. The diagram below (Figure 1) illustrates the different parts of the study and at what point the mixing of the data will occur. Following completion of the data collection and analysis of both datasets, the results will be merged. The quantitative and qualitative data will then be compared for convergence and divergence.

### Part 1: Questionnaire

Quantitative data from a questionnaire will be collected and analyzed (Multimedia Appendix 1). The questionnaire was developed in collaboration with a PPI group, GenerationR, at Alder Hey Hospital, Liverpool, United Kingdom. The PPI group gave us insight into: (1) the features required to create an accessible questionnaire design, (2) the decision-making processes involved in assessing orthodontic risk and benefit, (3) the personal responsibility attributed to maintain oral hygiene, and (4) the value and importance of pretreatment oral hygiene instruction using accessible methods.

The questionnaire is in 2 sections and includes 5 questions about participant information and 15 questions about participants’ perceptions of WSLs. A 5-point Likert-type scale will be used to assess participants’ perceptions of WSLs. Although there are limited studies assessing young people’s/parents’ perception of WSLs in orthodontics using a Likert-type scale, other published studies in orthodontics have used similar approaches and sample sizes to those proposed in our protocol [18]. Close-ended questions have been used to elicit higher response from participants (Multimedia Appendix 1) [19]. Participants will also be asked to rate a selection of images of WSLs of varying levels of severity before and after treatment.

### Part 2: Interviews

Qualitative data from one-to-one interviews will be collected to explore the findings from the questionnaire in more detail (Multimedia Appendix 2).

The study will use deductive (from the questionnaire responses) and inductive thematic analysis in a modified framework analysis approach that incorporates aspects of grounded theory [20]. The framework method will be used to gather data from the interviews for themes. It will follow Braun and Clarke’s 6-step framework [21]:

1. Familiarization of the data.
2. Create codes.
3. Generate themes.
4. Review themes.
5. Define themes.
6. Write up the results.

Main themes and subthemes will be developed iteratively alongside further data collection, with a search for confirming and disconfirming cases, until data saturation is reached [22].

### Research Team

AOH is the lead for the research. He is currently an orthodontic specialty trainee in the United Kingdom and is undertaking this project as part of a PhD degree. He has previous experience completing qualitative research and has an MRes degree.

JH is a professor and consultant in orthodontics in Liverpool, United Kingdom, and has a PhD degree. She is the Chief Investigator and primary supervisor for the project. She is currently involved in a randomized controlled trial looking at preventing WSLs (FL_4_OWS or Fluorides for Orthodontic White Spots), for which she has a research grant.

JD has experience in PPI and mixed methods research [23]. She has a PhD degree and has highlighted the importance of this study with respect to oral health–related stigma and shame and is cosupervising the project.

Amy Rawsthorne, who is not included as an author of this paper, is a research nurse who has previous experience in completing qualitative research. She will be assisting with recruitment, obtaining consent, completion of the questionnaires, and one-to-one interviews.

As AOH is the lead for this study and will be recruiting participants and interviewing them, none of the participants in this study will be treated by him as it may influence their responses.

The University of Liverpool has a strong history of caries research in relation to orthodontic treatment including in vitro, in situ, and case control studies, as well as randomized controlled trials [24-26].

### Context

The study will be undertaken in the orthodontic department at Liverpool University Dental Hospital, United Kingdom.

### Sampling Strategy

#### Inclusion Criteria

Individuals were recruited if they met the following criteria:

- Young people aged 11‐15 years inclusive who are considering or undergoing fixed orthodontic treatment at Liverpool University Dental Hospital, United Kingdom.
- Parent/guardian or person who has parental responsibility for a young person considering or undergoing fixed orthodontic treatment at Liverpool University Dental Hospital, United Kingdom.

#### Exclusion Criteria

Exclusion criteria were young people with:

- Learning difficulties that preclude them from answering the questionnaire or making an active contribution to interviews
- Craniofacial or other syndromes
- Nonorthodontic WSLs

For Part 1, the survey respondents will be recruited by convenience sampling methods from patients attending the orthodontic clinic at Liverpool University Dental Hospital, United Kingdom.

For Part 2, purposive sampling will be used to ensure the qualitative phase of the research recruits a sample with heterogeneous characteristics. Sampling will be based on age, gender, ethnicity, stage of treatment, and condition of first molar teeth based on previous clinical records (Table 1).

**Table 1.** Interview purposive sampling framework.^a^

		Gender	
		Male	Female
Functional appliance		Minimum 1	Minimum 1
Age (years)			
	11‐13	Minimum 1	Minimum 1
	14‐15	Minimum 1	Minimum 1
Ethnicity			
	Not Caucasian	Minimum 2	Minimum 2
Stage of treatment			
	Early	Minimum 1	Minimum 1
	Midtreatment	Minimum 1	Minimum 1
	Treatment completed	Minimum 1	Minimum 1
Condition of one or more first molars			
	Sound	Minimum 1	Minimum 1
	Restored/carious	Minimum 1	Minimum 1
	Extracted	Minimum 1	Minimum 1

a A minimum of 12 interview participants are needed.

#### Justification for This Framework

The sampling framework has been selected to represent a diverse sample and to limit bias. Differences condition of first molars are also considered because previous research within the department has shown that participants who experience WSLs have first molars in worse condition [27]; therefore, it would be useful to also collect participant data from this demographic as they are a higher risk group.

Data about the condition of first molars will be assessed using patient records and notes; participants will not be assessed by looking in their mouth or through taking radiographs specifically for this project.

#### Invitation

**Figure 2.**
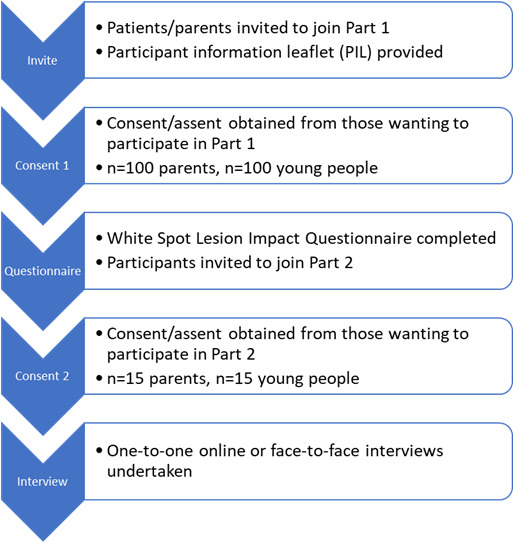
Participant journey during the study.

Eligible participants will be invited to take part in the research by a member of the research team (AOH). Eligible young people and their parents/guardians will be invited to complete the questionnaire on the same day as their routine orthodontic appointment. They will have free choice over whether they wish to take part and will be able to read the participant information leaflet during their appointment or after. Should they wish to take part in the study, they will be reimbursed for their time with a £10 (US $13.57) electronic voucher for the questionnaire and £25 (US $33.91) for the interviews. The flow of participants through the study is illustrated in Figure 2.

#### Sample Size

For Part 1, a pragmatic estimation of a sample size of 200 survey respondents (100 patients and 100 parents) had been initially agreed upon to address the aims and objectives of this part of the study. For a representative sample of patients aged 11‐15 receiving routine orthodontic treatment at the department, with a population of approximately 550 patients, 95% confidence level, and error margin of 5%, 226 respondents will be needed. Once data collection is near completion, the authors will ask a statistician to determine whether 226 respondents are needed.

For Part 2, for the qualitative research, 30 interviews with 15 young people and 15 parents/guardians will be undertaken. This sample size may be adjusted if data saturation is achieved earlier or not achieved and is comparable with the sample sizes of similar orthodontic studies [19,20].

### Ethical Considerations

Ethical approval was granted, via the Integrated Research Approval System (333499), on November 8, 2024. Sponsor Permission to Proceed notification was received from University of Liverpool (UoL001871) on January 30, 2025. Recruitment started on February 2, 2025. Participants will have free choice over whether they wish to take part and will be able to read the participant information leaflet during their appointment or after. Patients willing to take part in the study will be provided with a password-protected tablet displaying an information leaflet about the questionnaire. The information can also be accessed using their own device via a QR code. For those willing to participate, written assent will be obtained from the participants under the age of 16 years, and written consent will be obtained from their parent/guardian. The main ethical issues are maintaining confidentiality and asking questions that might be of a sensitive nature to young people and their parents/guardians. These will be addressed by strict compliance with institutional protocols about anonymization and storage of data. The young people will be interviewed separately from their parents/guardians to avoid corroboration. A chaperone will be present during the interviews with the young people, if requested. Participants will be able to stop or pause the interview at any stage. Should individuals wish to take part in the study, they will be reimbursed for their time with a £10 (US $13.57) electronic voucher for the questionnaire and £25 (US $33.91) for the interviews.

### Data Collection Methods, Instruments, and Technologies

#### Part 1: Quantitative Survey

Data collection for the survey started on February 2, 2025, and will continue until the sample size is met. Participant demographic data and the responses to the questions will be collected.

The participants will complete the questionnaire on a password-protected tablet in a private room near the orthodontic clinic at Liverpool University Dental Hospital. The questionnaire will be accessed using JISC Online Surveys through the University of Liverpool. Data will be transferred from the questionnaire using JISC and exported to an Excel 2018 (Microsoft Corp) spreadsheet for analysis.

The research team will review the data after 25% of the sample has completed the questionnaire to check if participants who have been recruited are representative of the demographics of the clinic. If any groups of people are underrepresented at this point, then this will be identified, and efforts will be made to recruit people from the underrepresented groups.

#### Part 2: Qualitative Survey

Data collection will start after Part 1 is complete and the data have been analyzed. It will continue until data saturation is achieved. Audio data will be transcribed and saved for analysis using NVivo software (version 15; Lumivero).

The young people will be interviewed separately from their parents/guardians to ensure discussions are not influenced by each other. If required, a chaperone will be present for the young people. A semistructured one-to-one interview process will enable the researcher to explore relevant topics raised by the participants.

The qualitative interviews will be conducted remotely via videoconference or in a private room in the orthodontic clinic at Liverpool University Dental Hospital, United Kingdom, using Teams. Audio recordings will be autotranscribed by Teams and checked and edited by a university-approved transcription service or AOH. Once transcribed, the audio recording will be deleted.

An interview guide/schedule has been devised to provide flexible direction and consistency and to probe all key topics sufficiently. The interview schedule will be adapted to take into account the findings of Part 1 of the study (Multimedia Appendix 2) and following a further PPI meeting to develop the interview schedule further. Discussions will last for approximately 45‐60 minutes. Interviews will continue until there are no recurring themes or patterns emerging from the data and thematic saturation has been achieved.

### Field Notes

Written notes will be taken throughout the research to document behaviors and communications that were not present in the audio. Field notes will also be useful to allow researchers to write reflections that will help in analyzing the data.

### Units of Study

The relevant inclusion and exclusion criteria will be applied as mentioned previously.

The target sample size will be 226 (113 patient and parent/guardian pairs) for Part 1 and 30 (15 patient and parent/guardian pairs) for Part 2 or until data saturation is reached.

### Data Processing

For Part 1, data from the questionnaire will be collected directly onto a password-protected tablet. Data will be transferred to and stored on a password-protected University of Liverpool or National Health Service computer during collection and for analysis. Data will be anonymized and analyzed using participant ID numbers. Microsoft Excel will be used for the descriptive data analysis.

For Part 2, interviews will be recorded directly onto a password-protected tablet. Transcription will be undertaken automatically via Teams and checked by a University of Liverpool provider or AOH. If needed, transcripts will be sent to participants for any clarification. NVivo software will be used to analyze the interview data.

Data will be retained in the University of Liverpool secure server for 10 years and deleted after. There will be no sharing of the data outside the immediate research team.

### Data Analysis

For Part 1, descriptive statistics will be used to analyze the data from the questionnaire. The κ statistic will be used to measure the agreement of responses between participants and their parents/guardians.

For Part 2, during the qualitative research analysis, data coding and identification of themes of transcripts will be undertaken by AOH using NVivo 12. The transcripts and codes/themes generated will be sent to a second or third researcher to confirm reliability (JH, JD, or AR). If needed, transcripts will be sent to participants for any clarification.

The study will use deductive (from the questionnaire responses) and inductive thematic analysis in a modified framework analysis approach that incorporates aspects of grounded theory [20]. The framework method will be used to gather data from the interviews for themes. It will follow Braun and Clarke’s 6-step framework [21]. Main themes and subthemes will be developed iteratively alongside further data collection, with a search for confirming and disconfirming responses, until data saturation is reached [15]. A coding tree will illustrate and organize the qualitative data to identify different themes.

Once both individual sets of data have been analyzed, they will be combined and compared for convergence or divergence, similar to another study published previously [28].

### Techniques to Enhance Trustworthiness

Data and methodological triangulation will be achieved by collecting data from a questionnaire and interviews to study the same phenomenon and allow researchers to cross-validate the findings. AOH will collect the data. JH and JD will be involved with data analysis and interpretation to ensure investigator triangulation. AOH will also maintain field notes of findings not recorded by the audio to allow for reflection.

To ensure member checking, the GenerationR PPI group will be involved in interpreting the Part 1 results and developing the interview schedule. Participants who have agreed to join this group will be contacted following data analysis to check whether they are happy with the interpretation and conclusions of the study and if they have any further comments to add. The research team will also present findings of the study to the PPI group to ensure their input into the interpretation of the findings.

AOH and AR will maintain an audit trail of the study documentation in line with Trust policy.

The research team will reflect on their own biases and assumptions throughout the research process and try to remain neutral during all stages of data collection, analysis, and interpretation. AOH will avoid recruitment of participants who are under his direct clinical care.

Peer review will be sought through presenting the results at local and national meetings prior to publication.

### Duration

A pilot of the study invited 10 young people and 10 parents/guardians to read the questionnaire and provide feedback on it. The authors were also able to monitor the length of time it took to invite and recruit people to the study. Following the pilot, an amendment to the time needed for recruitment was submitted to the sponsor and National Health Service ethics committee to allow for enough time for data collection for both parts of the study. A Gantt chart was created to provide further details on the duration of the study (Table 2).

**Table 2.** Gantt chart illustrating timelines for this white spot lesion research.

	Jan-25	Feb-25	Mar-25	Apr-25	May-25	Jun-25	Jul-25	Aug-25	Sep-25	Oct-25	Nov-25	Dec-25	Jan-26	Feb-26	Mar-26
Recruitment for quantitative research	✓	✓	✓	✓	✓	✓	✓	✓							
Data collection for quantitative research	✓	✓	✓	✓	✓	✓	✓	✓							
Data analysis of quantitative research								✓							
Recruitment for qualitative interviews									✓	✓	✓	✓	✓	✓	✓
Transcription of interviews									✓	✓	✓	✓	✓	✓	✓
Data analysis of interviews												✓	✓	✓	✓

## Results

### Progress to March 13, 2025

Funding for the study was secured through a joint Faculty of Dental Surgery and Royal College of Surgeons of England/British Orthodontic Society pump-priming grant in May 2024. Ethical approval was granted via the Integrated Research Approval System (333,499) on November 8, 2024. The Sponsor Permission to Proceed notification was received from the University of Liverpool (UoL001871) on January 30, 2025. Recruitment started on February 2, 2025. As of August 31st, 2025, seventy five participant pairs have been recruited. Results are expected to be published in a peer-reviewed journal in the summer of 2026.

### Part 1

Demographic data of the sample and descriptive data for the responses from the questionnaire together with associations between participants and the young people and parent/guardian pairs will be presented.

### Part 2

Themes and subthemes will be explored; interpretations and inferences will be presented, including links to empirical data from the interview transcripts.

## Discussion

### Overview

In the final paper, a summary of the findings will be presented. The results will be compared to the existing literature. The strengths and limitations of the study and data will be explored. The implications for clinical practice and further research will be discussed.

### Strengths

The aims of orthodontic treatment are to improve the occlusion and appearance of teeth, which benefits function, confidence, self-esteem, and quality of life [23,29]. Detecting WSLs after removing fixed orthodontic appliances may detract from the benefits of orthodontic treatment. WSLs are likely to have negative associations for people with anterior tooth discoloration, and they may experience unfavorable judgments of personality traits and characteristics, which may affect friendships, relationships, and career prospects [30].

WSLs also have implications for the consent process for orthodontic treatment. Although young people can understand risks, they can have false perceptions about them if they are not fully understood [31]. Poor communication can lead to issues with consent and other negative outcomes like complaints and litigation [31].

This mixed methods study has the potential to inform clinicians’ communication about WSLs with young people and their parents/guardians. Furthermore, the study may help researchers improve their understanding of what methods can help to inform young people of the potential consequences of WSLs and support WSL prevention. Even with effective oral hygiene instruction, around half of young people do not follow the clinician’s advice to improve their oral hygiene [12]. The COM-B model is presented as a tool to diagnose which of capability, opportunity, or motivation need to change for a new behavior to take place [32]. Although interventions designed to improve oral hygiene during orthodontic treatment (including using smartphones and a toothbrushing app, visual aids, motivational interviewing, oral health reinforcements) have been investigated, only the use of mobile phones has had limited evidence for improving oral health during orthodontic treatment [33]. To our knowledge, studies have not been undertaken to explore barriers to oral hygiene or behavioral interventions to reduce WSL formation during orthodontic treatment in young people.

### Limitations

The study is only recruiting participants from one hospital in Liverpool, United Kingdom, and there may be a difference if the young people and parents/guardians were to be recruited in primary care or from another area of the United Kingdom and/or another country. Patients attending hospital orthodontic departments tend to have more complex treatment needs, which might influence their perceived posttreatment satisfaction.

With all cross-sectional studies, there are limitations to questionnaires, as they collect data at a set time point and are therefore unable to establish cause and effect. The participants who are likely to respond to the questionnaire are more likely to be young people and their parents/guardians who are interested in the project and may be more motivated to prevent orthodontic WSLs than those who do not volunteer to take part. Although it will be difficult to ensure all relevant groups in the population are included, the study will recruit a heterogeneous sample of participants with regard to age, gender, condition of first molars, and type of orthodontic treatment they are receiving. The study will identify participants from different cells of a sampling framework (Table 1). Social desirability bias has been limited by asking participants not to discuss answers with parents/guardians as this may influence their answers. The participants are able to complete the questionnaire in a private room without a researcher being present. The study will not recruit any participants who are under the clinical care of the research team involved in recruiting. PPI will be used throughout all stages of the research to ensure questions are relevant to the participants and not misleading.

As a clinician/dentist, AOH will undertake the qualitative research; this increases the risk that the researcher could make assumptions about what the participants think or feel based on their professional experience. Parents/guardians may not want to feel blamed by health care professionals and may want to avoid being responsible for the young person’s poor oral hygiene practices. Participants may forget to recall information during the interview, and all participants could answer questions differently based on what they think the researcher would like to hear as the “correct” answer rather than discussing their own honest experience, especially if they are aware that the researcher is a clinician. As discussed, the study will confirm the reliability of the answers to the questionnaire and qualitative interviews. To aid transparency, if there is a disagreement in the interpretation of the qualitative data, the themes/codes will be sent to another researcher for secondary/tertiary analysis. Where possible, AOH will avoid recruiting patients directly under his care, as it may affect their responses and/or his questions due to prior knowledge of each other. The study will also promote reflexivity through a postinterview debrief with participants and members of the research team. This will help to ensure that the study findings agree with the participant views rather than any subjectivity or researcher bias. Participants also can review study findings to ensure that they agree with the results. The authors have also attempted to address self-reporting bias by publishing the study protocol, the questionnaire/interview schedule, and the data so that readers are able to make an informed decision about the potential sources of bias.

### Conclusions

This is a mixed methods study that aims to investigate the impact of WSLs on young people who are considering or undergoing orthodontic treatment with fixed appliances and their parent/guardian, as well as to explore their perceptions, attitudes, and feelings toward WSLs.

## Supplementary material

10.2196/60213 Multimedia Appendix 1 Questionnaire.

10.2196/60213 Multimedia Appendix 2 Interview schedule.

## Abbreviations

FA: fixed appliance
PPI: patient and public involvement
WSL: white spot lesion
