# Supplementary material for: Perception and Impact of White Spot Lesions in Young People Undergoing Orthodontic Treatment and Their Guardians: Protocol for a Mixed Methods Study
Source: JMIRx Med. 2025 Sep 12;6:e60213. doi: 10.2196/60213 (PMC12431786; doi:10.2196/60213)
Supplement: Multimedia Appendix 1 [file xmed-v6-e60213-s001.docx]

Questionnaire (draft
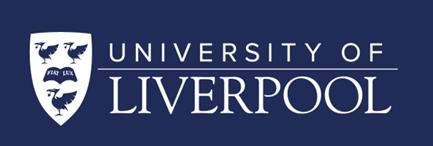
)

WHITE SPOT LESION IMPACT QUESTIONNAIRE

[YOUNG PEOPLE OR PARENTS/GUARDIANS]


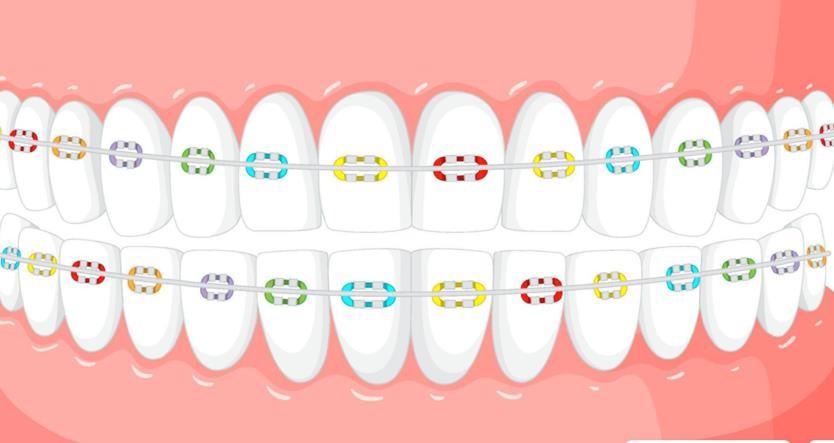


**Part 1 - Participant information**

| Question 1. |  |
| --- | --- |
| What is your name? |  |

| Question 2. |  |
| --- | --- |
| What is your date of birth? |  |

| Question 3. |  |
| --- | --- |
| What is your parent/guardians or child’s name? |  |

| Question 3. |  |
| --- | --- |
| With which gender do you identify? | - Male - Female - Other (please state) - I’d rather not say |

| Question 4. |  |
| --- | --- |
| What is your ethnicity? | - White British - Any other white background - Indian, Pakistani or Bangladeshi - Asian - African - Caribbean - Arab/middle eastern - Mixed race (please state) - Rather not state - Other (please state) |

| Question 5. |  |
| --- | --- |
| At what stage of treatment are you / your child? | - Thinking about having brace treatment. - Wearing a brace - Had brace taken off |

**Part 2 – Participant Questionnaire**

**PLEASE SELECT ONLY ONE OPTION THAT BEST DESCRIBES YOUR ANSWER**

| Question 1. |  |
| --- | --- |
| Do you remember being told that white marks could form on teeth during brace treatment? | Please write any thoughts you have about this, below |
| - Yes - No - I’m not sure |  |

| Question 2. |  |
| --- | --- |
| What did you think of the conversation you had about white marks forming on teeth during brace treatment? | Please write any thoughts you have about this, below |
| - It was explained very well. - It could be explained better. - I was given some information, but it wasn’t enough. - I wasn’t given any information. - I’m not sure |  |

| Question 3. |  |
| --- | --- |
| Do you know why people get white marks on their teeth after brace treatment? | Please write any thoughts you have about this, below |
| - Not brushing properly. - Drinking acidic drinks (like fizzy drinks   / fruit juice)   - Eating sugary snacks - A mix of not brushing, eating sugary snacks and drinking acidic drinks - I’m not sure |  |

| Question 4. |  |
| --- | --- |
| Which problem bothers you the most after brace treatment? | Please write any thoughts you have about this, below |
| - Brown marks on front top teeth - White marks on front top teeth - Root shortening of teeth - Gum shrinkage (recession) - Teeth moving back to where they were before braces were fitted (relapse) |  |

| Question 5. |  |
| --- | --- |
| How likely do you think you/ your child will get white or brown marks on your/ their teeth | Please write any thoughts you have about this, below |
| - Very likely - Quite likely - Likely - Unlikely - Very unlikely |  |

| Question 6. |  |
| --- | --- |
| How worried are you about having white marks on your / your child’s teeth after brace  treatment? | Please write any thoughts you have about this, below |
| - Extremely worried - Quite worried - A little worried - OK - Not worried |  |

| Question 7. |  |
| --- | --- |
| How would you feel if you had marks on your teeth, at the end of treatment, like in the photo? | Please write any thoughts you have about this, below |
| - Very Happy - Happy - OK - Unhappy - Very Unhappy |  |
| 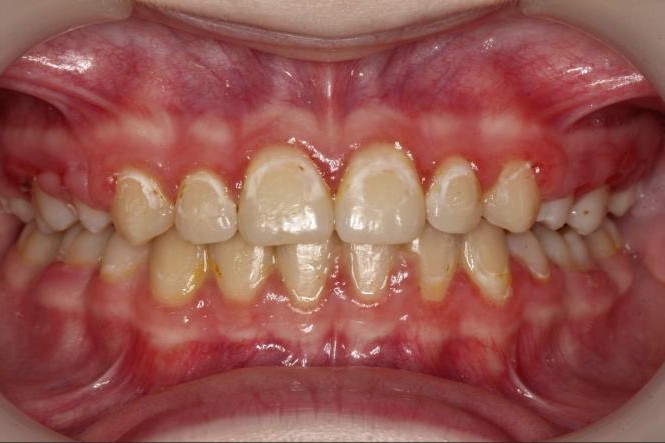 | |

| Question 8. |  |
| --- | --- |
| How would you feel if you had teeth before (on the left) and after (on the right) after braces? | Please write any thoughts you have about this, below |
| - Very Happy - Happy - Ok - Unhappy - Very Unhappy |  |
| 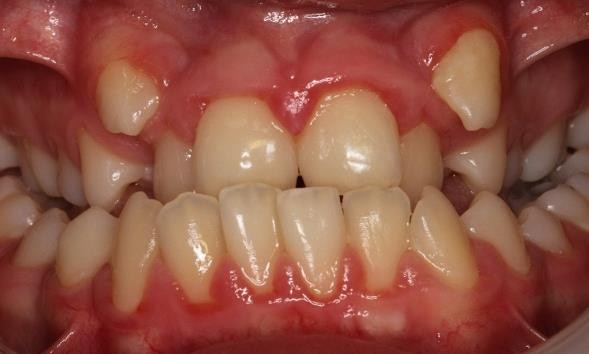 **8. Before**  **8. Before** | 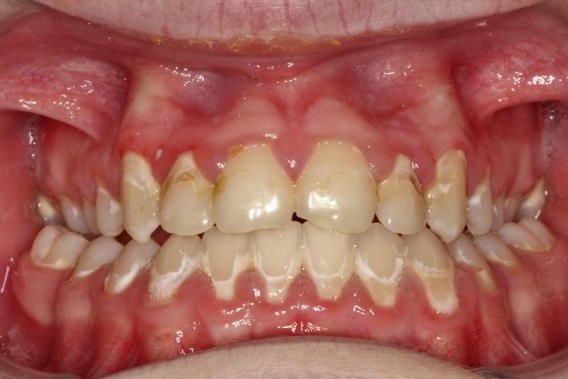 **8. After** |

| Question 9. |  |
| --- | --- |
| How would you feel if you had teeth before (on the left) and after (on the right) after braces? | Please write any thoughts you have about this, below |
| - Very Happy - Happy - Ok - Unhappy - Very Unhappy |  |
| 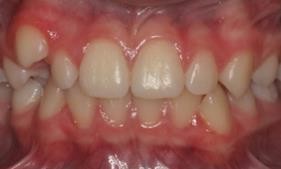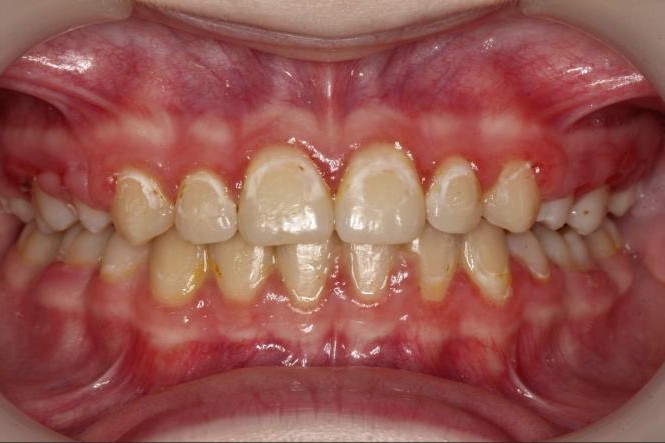 **9. Before**  **9. After** | |

| Question 10. |  |
| --- | --- |
| How would you feel if you had teeth before (on the left) and after (on the right) after braces? | Please write any thoughts you have about this, below |
| - Very Happy - Happy - Ok - Unhappy - Very Unhappy |  |
| 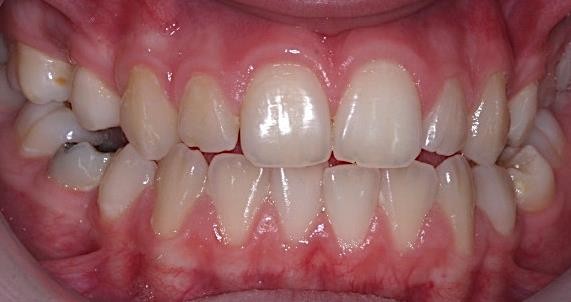 **10. Before**  **10. Before** | 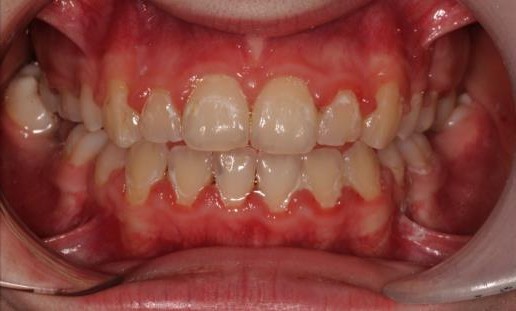 **10. After**  **10. After** |

| **Question 11.** | |  |
| --- | --- | --- |
| Rank in order what you think you or your child would be willing to do to prevent white/brown marks (1-5, 1= most preferred option, 5=least) | | Please write any thoughts you have about this, below |
|  | Rub a pea sized blob of toothpaste on my teeth every night before bed |  |
|  | Visit the dentist every 3 months to place varnish during a dental check-up |  |
|  | Let the orthodontist place a varnish on my teeth during my brace visits |  |
|  | Rinse daily with mouthwash at a separate time to brushing |  |
|  | Spend time brushing teeth, cleaning in between teeth with bottle brushes and cut out sugary foods and drinks |  |

| Question 12. |  |
| --- | --- |
| How easy was it for you to understand this questionnaire? | Please write any thoughts you have about this, below |
| - Very easy - Easy - OK - Difficult - Very difficult |  |

| Question 13. |  |
| --- | --- |
| Do you feel the questionnaire asked questions about white spots, were important to young people and parents /  guardians, like you? | Please write any thoughts you have about this, below |
| - Yes - No - Not sure |  |

| Question 14. |  |
| --- | --- |
| Would you like the research team to contact you about the study findings? | - Yes - No |

| Question 15. |  |
| --- | --- |
| Would you like to take part in the second part of the study which includes a chat?  If you do wish to take part, then please note that you may not be selected for interview. | - Yes - No |
