# Supplementary material for: Perception and Impact of White Spot Lesions in Young People Undergoing Orthodontic Treatment and Their Guardians: Protocol for a Mixed Methods Study
Source: JMIRx Med. 2025 Sep 12;6:e60213. doi: 10.2196/60213 (PMC12431786; doi:10.2196/60213)
Supplement: Multimedia Appendix 2 [file xmed-v6-e60213-s002.docx]

**Introduction about the study and interview**

**Knowledge and beliefs of white spot lesions**

What do you know about white marks on teeth after brace treatment (icebreaker question)

Do you/parent think you do enough to avoid getting white marks on your teeth after brace treatment?

Whose responsibility is it to avoid getting white marks on your teeth?

What can the dental team do to help you / your son/daughter avoid white spot lesions?

If you had white spot lesions on your teeth:

1. How do you think they would affect you?
2. How would manage them?

Tell me about a time you remember where white marks were mentioned as part of brace treatment?

1. How much of a problem do you think white spot lesions are to you during brace treatment?
2. How much of a problem do you think white spot lesions are to other people who have brace treatment?

What is the best way to discuss risks of white marks during brace treatment between clinician and patient/parent?

- Probes: how are white spot lesions formed? How much do you think white marks are likely to affect your brace treatment? How common do you think white marks are at the end of brace treatment? What sort of discussion did you have about white spot lesions before you started treatment? E.g., verbal/visual/once/repetitive? What other ways could staff motivate you/other patients to avoid white marks Whose responsibility (Parent/young person/clinician) is it to avoid/manage white spot lesions?

**Attitudes towards WSL**

To what degree do you think that these white marks would stop you from doing your usual activities like socialising, smiling etc.? (mild / mod / severe images)

How likely would you be to make a verbal/written complaint about white marks following brace treatment? (mild/mod/severe)

Would this change if the marks were brown?

How else might you be affected if you had white marks on your teeth?

In terms of overall risks of brace treatment, how much did white spot lesions bother you about brace treatment compared to other risks?

How common did you think about/worry about white marks during brace treatment?

Who do you think should pay the treatment of the white spots?

- Probes:
  - How did you feel about white spot lesions before this study?
  - Tell me about your worries of WSL from brace treatment.
  - How sever do you think the changes are? – mild/moderate/severe, brown/white/pitted/front teeth/back teeth/small/large?
  - Would have they stopped you from having brace treatment?
  - What differences do you think clinicians/patients/parents have about their views of white marks?
  - Do you think the term white spot lesions accurately describes the risk it poses?
  - Who do you think should pay for the treatment of these marks? – the patient / GDP / orthodontist?

**Motivation towards avoiding WSL.**

How did having brace treatment affect the way you look after your teeth (to YP)? (Rephrase depending at what stage the young person is in treatment pre/mid/post)

How does your son/daughter having brace treatment affect the way you helped with his/her brushing/mouth care of the young person because of brace treatment (to parent)? (Rephrase depending at what stage the young person is in treatment pre/mid/post)

How did knowing your son/daughter was having brace treatment affect the way you supported the oral health of your young person because of brace treatment (to parent)?

How you feel (patient/parent) if you were told that the young person’s tooth brushing/diet is not very good meaning that there is a higher chance of him/her developing/getting white marks?

Why do you think a young person will find it difficult to respond to warnings about oral health and risks of WSL?

1. what do you think clinicians can do to help avoid white marks for patients?
2. Who do you think is best at talking to patients about this – person doing the brace treatment / their general dentist / a dental nurse/hygienist?

What can clinicians do to help prevent people from getting white spots?’

(Same questions again but what can patients/parents do to avoid getting white marks)

How do you feel long brace treatment (2-3 years) affects a person’s motivation to look after their teeth?

How would you feel about having brace treatment if you knew you would develop a severe white mark?

- Probes:
  - What are the barriers to avoiding WSL (patients’ parents’)?
  - Do you clean your teeth any differently since you started brace treatment? (e.g., ways to clean under brace/interdental brushes).
  - Do you find that knowing about the risks of white marks, compared to other problems of brace treatment, has changed the way you looked after your teeth?
  - How can clinicians motivate young people to improve toothbrushing/diet to avoid WSL (e.g., videos, leaflets, pictures)?
  - Would having marks on your teeth like this……. make you think twice about having brace treatment? or similar.

**Summary**

Summarise the young person’s / patent/guardian’s thoughts and perceptions about WSL.

**Thank you**

Thank the participants for taking part in the study and let them know the results.
